# *Rana grylio* Virus (RGV) 50L Is Associated with Viral Matrix and Exhibited Two Distribution Patterns

**DOI:** 10.1371/journal.pone.0043033

**Published:** 2012-08-13

**Authors:** Xiao-Ying Lei, Tong Ou, Qi-Ya Zhang

**Affiliations:** State Key Laboratory of Freshwater Ecology and Biotechnology, Institute of Hydrobiology, Chinese Academy of Sciences, Graduate School of the Chinese Academy of Sciences, Wuhan, China; University of Kansas Medical Center, United States of America

## Abstract

**Background:**

The complete genome of *Rana grylio* virus (RGV) was sequenced and analyzed recently, which revealed that RGV 50L had homologues in many iridoviruses with different identities; however, the characteristics and functions of 50L have not been studied yet.

**Methodology/Principal Findings:**

We cloned and characterized RGV*50L*, and revealed 50L functions in virus assembly and gene regulation. *50L* encoded a 499-amino acid structural protein of about 85 kDa in molecular weight and contained a nuclear localization signal (NLS) and a helix- extension-helix motif. Drug inhibition assay demonstrated that *50L* was an immediate-early (IE) gene. Immuno-fluorescence assay revealed that 50L appeared early and persisted in RGV-infected cells following two distribution patterns. One pattern was that 50L exhibited a cytoplasm-nucleus- viromatrix distribution pattern, and mutagenesis of the NLS motif revealed that localization of 50L in the nucleus was NLS-dependent; the other was that 50L co-localized with viral matrix which plays important roles in virus assembly and the life circle of viruses.

**Conclusions/Significance:**

RGV *50L* is a novel iridovirus IE gene encoded structural protein which plays important roles in virus assembly.

## Introduction

*Rana grylio* virus (RGV) is a pathogenic agent that causes lethal disease in cultured pig frogs (*Rana grylio*), which was the first iridovirus isolated in China [1], [2]. Previous studies have revealed that RGV is a large, icosahedral, dsDNA virus, belonging to the family *Iridoviridae* and closely related to frog virus 3, the type species of the genus *Ranavirus* [3]–[5]. At least 16 structural proteins were detected [2]. Cellular changes and some viral proteins involved in RGV infection and replication have been identified and characterized, such as 3β-hydroxysteroid dehydrogenase (3β-HSD), deoxyuridine triphosphatase (dUTPase), thymidine kinase (TK) and a gene belonging to the essential for respiration and viability family (ERV1) [6]–[11]. In application, a recombinant RGV containing EGFP gene (ΔTK-RGV) was constructed, which could be easily detected by fluorescent microscopy [12]. Recently, the complete genome of RGV has been sequenced and analyzed, and the results confirmed that RGV belongs to the genus *Ranavirus* [13].

Iridoviruses, belonging to Nucleo-Cytoplasmic large DNA viruses (NCLDVs), contain circularly permutated and terminally redundant double-stranded DNA genomes ranging from 103 to 212 kbp in length and replicate in both the nucleus and cytoplasm of infected cells, and could infect varieties of invertebrates and poikilothermic vertebrates [14]. Based on the Ninth Report of the International Committee on Taxonomy of Virus (ICTV), the family *Iridoviridae* is currently classified into five genera: *Ranaviru*s, *Lymphocystivirus, Megalocytivirus*, *Iridovirus* and *Chloriridovirus* [15]. Members of the genus *Ranavirus* could cause systemic disease or die-offs in a wide range of economically and ecologically important vertebrates including fish, amphibians and reptiles, which have become serious problems in modern aquaculture, fish farming and wildlife conservation, leading to serious economic losses [16]–[18].

Virion assembly of iridoviruses takes place in electron-lucent viral matrix (virus factory) which contains virus particles at different stages of assembly, including empty capsids, capsids with partial cores and the matured nucleocapsids [3], [5]. Little is known about the precise process of virion morphogenesis in iridoviruses. Up to date, only two structure proteins of iridoviruses have been identified to be linked to virion assembly, including the major capsid protein (MCP) (RGV ORF 97R) and a putative myristoylated membrane protein (ORF 53R of RGV and FV3) [19]. MCP of iridovirus is an internal lipid membrane, the sequence of which is highly conserved within all members of the family [20]. The MCP comprises 40% of the total virion protein content and contains the viral genome, constituting the inner core of iridovirus particles [21], [22]. Knock down studies using artificial microRNAs and asMOs demonstrated that 53R was indispensable for virion assembly, and *in* *vitro* studies also showed that 53R was associated with virus factories and the virion membrane [23]–[25].

**Figure 1:**
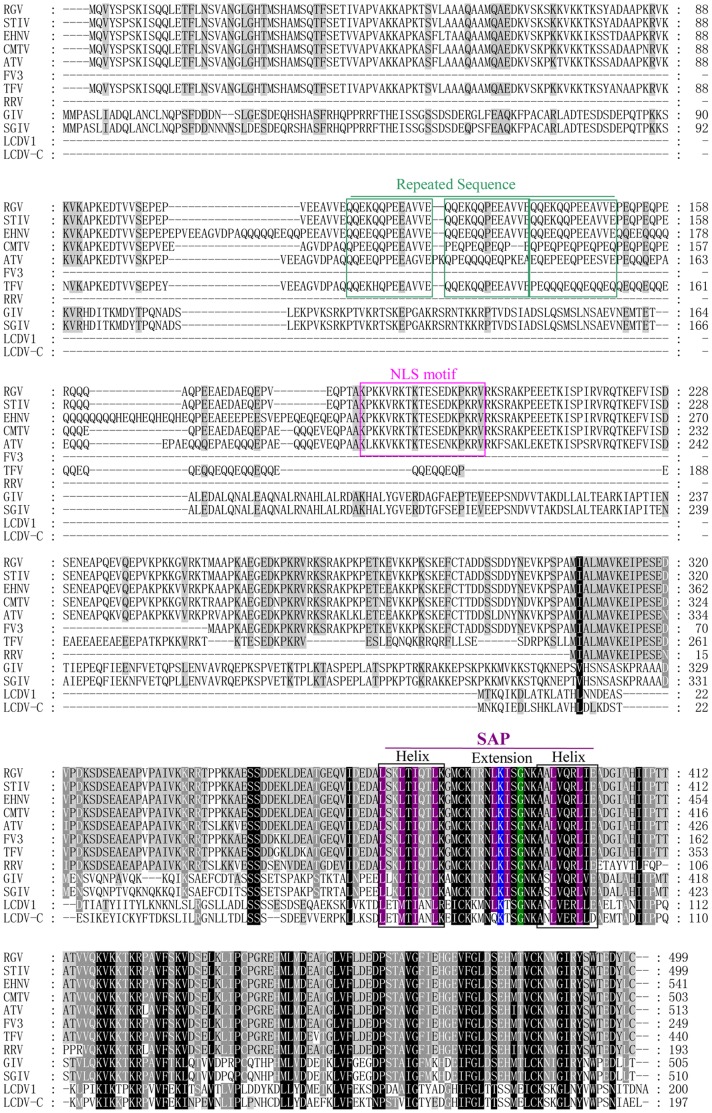
Multiple sequence alignment of 50L homologues in iridoviruses. RGV, *Rana grylio* virus; STIV, soft-shelled turtle iridovirus; CMTV, common midwife toad ranavirus; EHNV, epizootic hematopoietic necrosis virus; ATV, *Ambystoma tigrinum* virus; TFV, tiger frog virus; FV3, frog virus 3; RRV, Regina ranavirus; GIV, grouper iridovirus; SGIV, Singapore grouper iridovirus; LCDV-1, lymphocystis disease virus 1; LCDV-C, lymphocystis disease virus-China. The completely conserved amino acid residues are indicated by a black background, while the grey background are partially conserved residues with greater than 80% identity, and key amino acid residues in SAP domain are indicated by colorful backgrounds. Conserved motifs are shown by rectangles and labeled as Repeated Sequence, NLS motif and SAP domain above the alignment, respectively. Gaps (dashes) were introduced to maximize the alignment.

Analysis of the RGV genome showed that it contains 106 ORFs encoding peptides ranging from 41 to 1294 amino acids in length, and the ORF 50L, containing a putative SAP motif [named after SAF-A/B, Acinus and PIAS (protein inhibitor of activated STAT)], shares high identity with soft-shelled turtle iridovirus (STIV) while relatively low with FV3 [13]. The homolog of RGV 50L in Singapore grouper iridovirus (SGIV), SGIV 25L, has been detected by LC-MALDI workflow [26], however, the characteristics and functions of the gene have not been studied yet.

**Table 1:** Comparisons of RGV 50L with its homologues in other iridoviruses.

Virus	Accession No.	ORF^a^	Length (aa)^b^	MW^c^ (kDa)	Id%^d^
RGV	JQ654586	50L	499	55.5	100
STIV	EU627010	52L	499	55.5	100
CMTV	JQ231222	59R	503	55.8	89
EHNV	FJ433873	83L	541	60.7	85
ATV	AY150217	79L	513	60.0	82
TFV*	AF389451	51L, 52L	440	49.5	69
FV3	AY548484	49L	249	27.5	49
RRV	–	–	193	21.4	33
GIV	AY666015	9L	505	55.8	23
SGIV	AY521625	25L	510	56.5	23
LCDV-1	L63545	59L	200	22.2	12
LCDV-C	AY380826	62R	197	22.3	12

To understand the role of RGV 50L in iridovirus propagation, we cloned RGV *50L* gene, prepared anti-RGV 50L serum, characterized its expression pattern and detected its molecular mass. Then, cycloheximide (CHX) and cytosine arabinofuranoside (Ara C) were used to identify the expression pattern of RGV *50L*. Subsequently, EGFP-50L and NLS motif mutant EGFP-50L-ΔNLS were constructed to identify subcellular locations of the fusion protein. Moreover, ΔTK-RGV and anti-RGV 50L serum were used to detect the localization of 50L protein during RGV infection. Furthermore, in order to know the effect of 50L on other RGV genes, real-time quantitative PCR of *MCP* were determined in 50L-pcDNA3.1 stably transfected cells.

**Figure 2:**
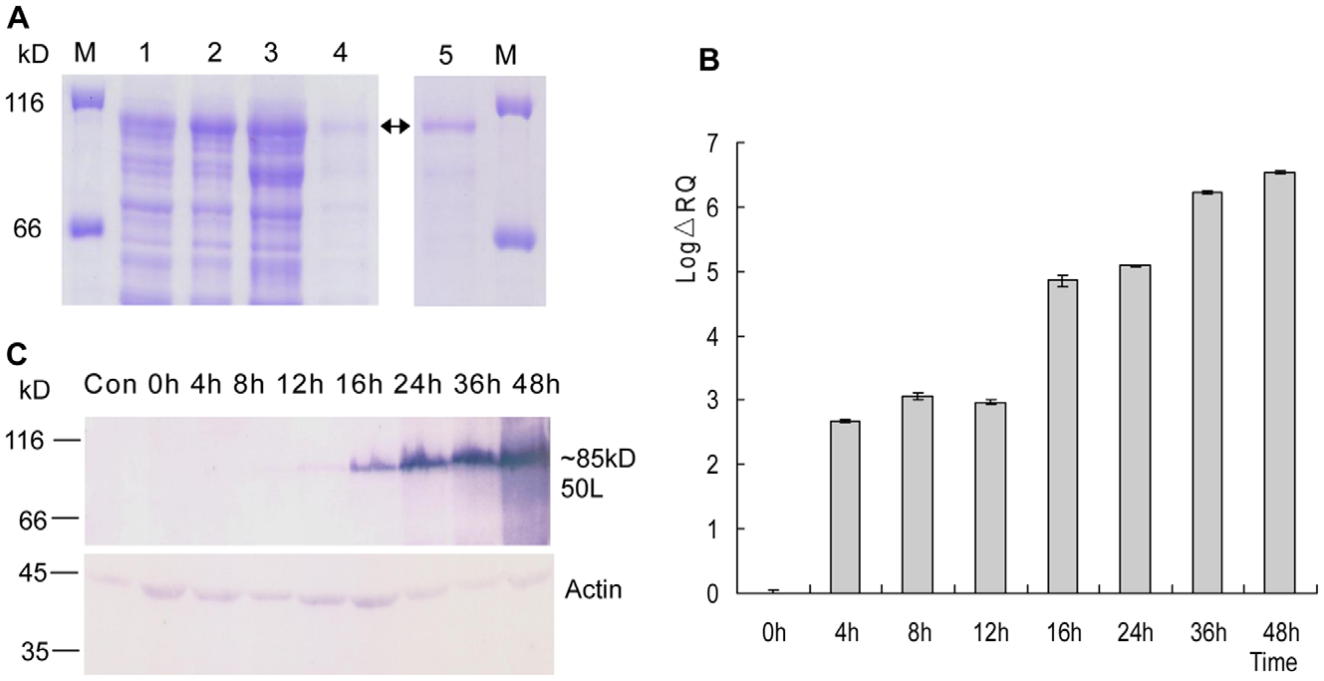
Prokaryotic and temporal expression of RGV 50L. (A) SDS-PAGE of prokaryotic expressed and purified fusion protein 50L-His. Lane 1: pET32a/50L, non-induced; lane 2: pET32a/50L, induced; lane 3: precipitate of induced pET32a/50L after ultrasonication; lane 4: supernatant of induced pET32a/50L after ultrasonication; lane 5: the purified protein by Ni^2+^-NTA affinity chromatography. (B) Real-time quantitative PCR detection of RGV transcriptional levels in RGV-infected EPC cells. EPC cells were infected by RGV at an M.O.I. of 1. 50L mRNA levels was measured by real-time PCR analysis at different time (0, 4, 8, 12, 16, 24, 36 and 48 h) post-infection (p.i.), mock infected cells was used as negative control. Transcriptional level of RGV 50L mRNA was expressed by the common logarithm of the relative quantity (Log ΔRQ). All the values were normalized to the β-actin gene. The values represent averages of three independent experiments, with the range indicated (±SD). (C) Western blot analysis of temporal expression pattern of 50L protein. Proteins from the experiment described in (B) were analyzed by western blot analysis, and β-actin was detected under the same conditions as an internal control. Protein markers were indicated (lane M).

## Results

### Sequence Analysis of RGV 50L

The complete ORF of RGV (GenBank Accession No. JQ654586) 50L, a fragment of 1500 bp in length, was amplified from RGV genomic DNA using specific primers. Sequence analysis revealed that RGV 50L encodes 499 amino acids and contains several conserved features, including a lysine-rich nuclear localization signal (NLS), a helix-extension-helix motif (putative SAP domain) and a continuous QQEKQQPEE AVVE tri-repeated sequence (Fig. 1). 50L had homologues in many iridoviruses, showing high identities (82∼100%) with STIV 52L, common midwife toad ranavirus (CMTV) 59R, epizootic hematopoietic necrosis virus (EHNV) 83L, and *Ambystoma tigrinum* virus (ATV) 79L, while relatively low (less than 50%) with FV3 49L, grouper iridovirus (GIV) 9L, Singapore grouper iridovirus (SGIV) 25L, lymphocystis disease virus (LCDV-1) 59L and lymphocystis disease virus-China (LCDV-C) 62R (Table 1).

**Figure 3:**
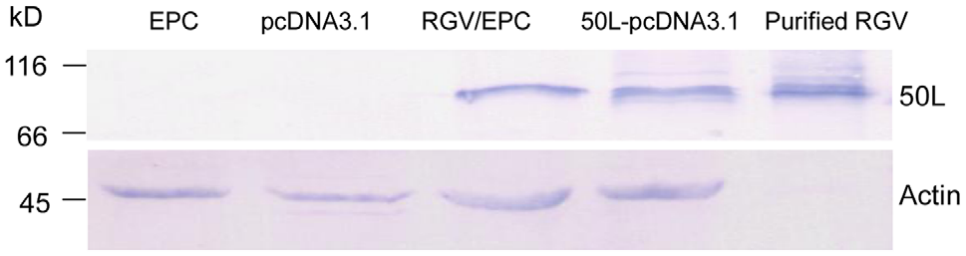
Molecular weight detection of 50L. EPC cells were mock (EPC), infected by 1 M.O.I. RGV (RGV/EPC), transfected with plasmid pcDNA3.1 (pcDNA3.1) and 50L-pcDNA3.1 (50L-pcDNA3.1), respectively, after incubated for 12 h, the samples were detected by western blot assay. The purified RGV particles were analyzed together (Purified RGV). Protein markers were indicated.

### Prokaryotic and Temporal Expression of RGV 50L

To prepare anti-RGV 50L serum, pET32a-50L was transformed into *Escherichia coli* BL21 (DE3) and expression of the 50L-His fusion protein was induced. As shown in Fig. 2A, the induced fusion protein was approximately 75 kDa (Lane 2–4), whereas no protein band was found at the same position of the non-induced 50L-pro/BL21 (Lane 1). The fusion protein was purified using Ni^2+^-NTA affinity chromatography (Lane 5), and used to prepare anti-RGV 50L antibody in mice.

**Figure 4:**
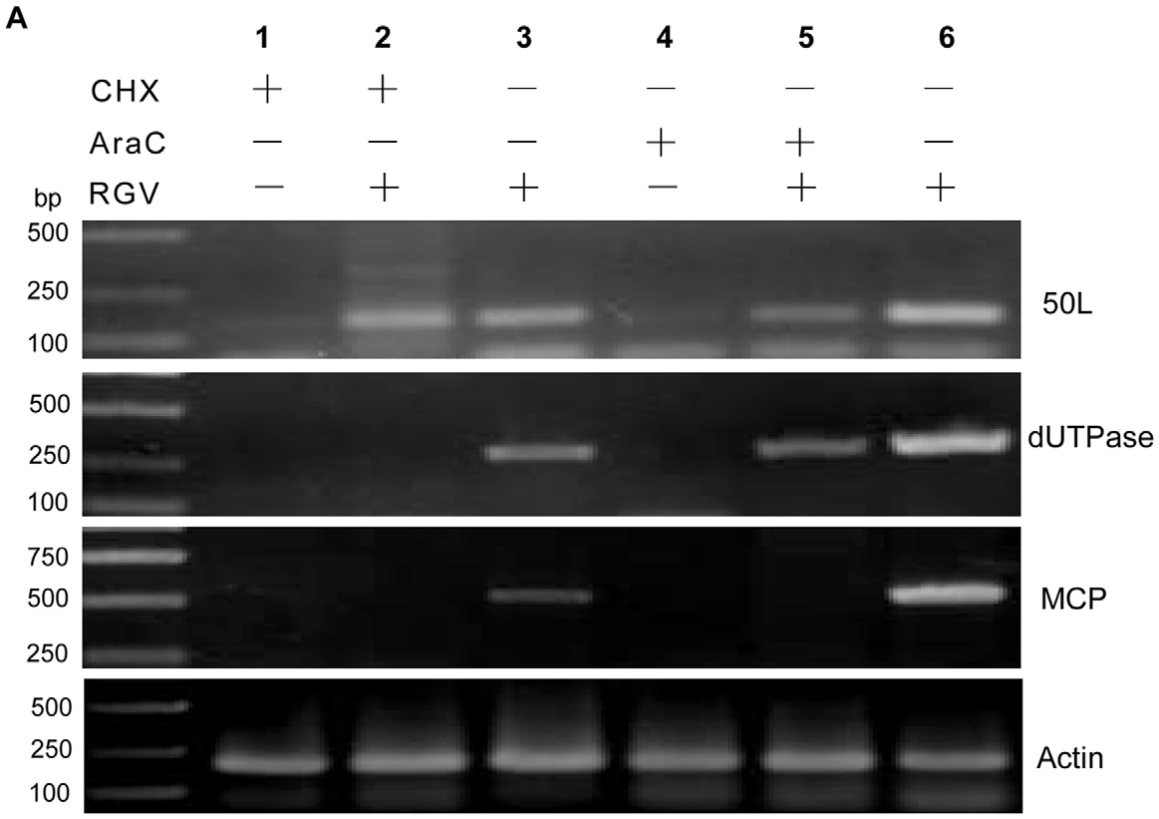
RT-PCR and western blot detection of 50L under drug treatments. (A) RT-PCR analysis of RGV 50L gene following treatments with CHX or AraC. Lane 1: CHX-treated uninfected at 6 h p.i.; lane 2: CHX-treated RGV-infected at 6 h p.i.; lane 3: RGV-infected at 6 h p.i.; lane 4: AraC-treated uninfected at 48 h p.i.; lane 5: AraC-treated RGV-infected at 48 h p.i.; lane 6: RGV-infected at 48 h p.i.; and DNA markers are indicated. Every sample was detected by RT-PCR using primers of 50L, dUTPase, MCP, respectively. β-actin gene was used as an internal control. (B) Western blot analysis of RGV 50L expression following treatments with CHX or AraC. Protein samples from described in (A) were analyzed by western blot analysis, and β-actin was detected under the same conditions as an internal control. Protein markers were indicated.

The temporal expression pattern of RGV 50L was characterized by real-time quantitative PCR (qRT-PCR) and western blot analysis. Transcriptional level of RGV 50L was expressed by the common logarithm of the relative quantity (Log ΔRQ). As shown in Fig. 2B, transcripts of 50L increased from 4 h post infect (p.i.) in RGV-infected cells and the value of Log ΔRQ was more than six at 48 h p.i. A specific protein band for 50L could be detected from 8 h p.i. by western blot assay using anti-RGV 50L antibody and the quantity also increased with the elongation of infection time (Fig. 2C).

**Figure 5:**
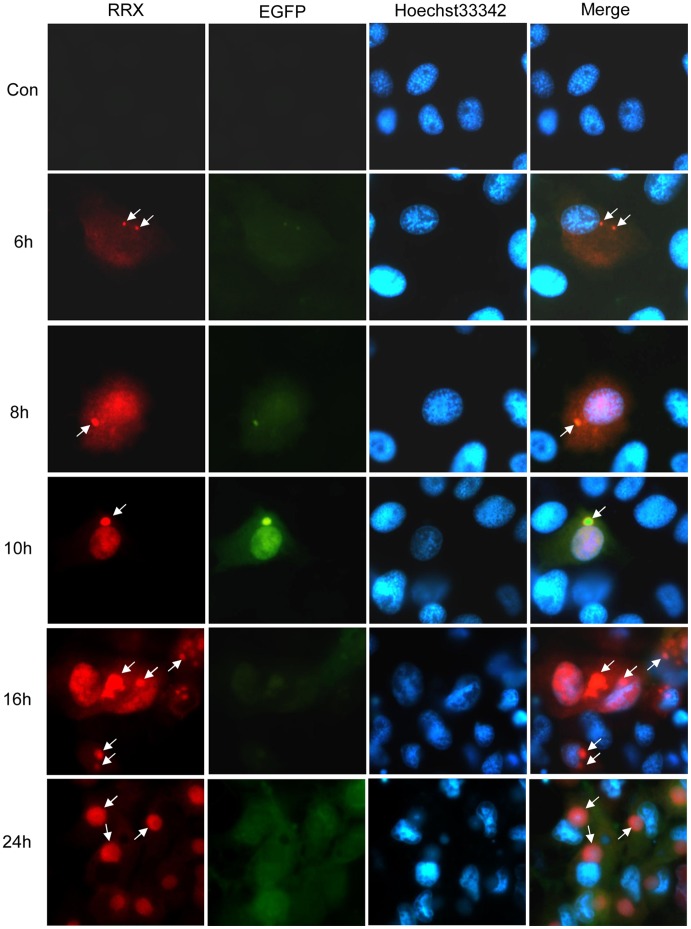
Immuno-fluorescence localization of 50L during RGV-infection. EPC cells were infected with 1 M.O.I. of ΔTK-RGV for 6, 8, 10, 12 and 24 h, and fixed, permeabilized and stained with anti-RGV 50L serum and RRX-conjugated anti-mouse antibody, followed by Hoechst 33342. Mock-infected cells were used as a negative control. Red fluorescence showed the localization of the fusion protein (RRX), green fluorescence showed the virus infected cells (EGFP), the cell nuclei were shown by Hoechst 33342 (Hoechst 33342), and the merged photos were also listed (Merge). The arrows indicated viral matrices. Magnification ×100 (oil-immersion objective).

### Molecular Mass Detection of RGV 50L

The MW of 50L shown in Fig. 2C was about 85 kDa, which was much larger than the data 55 kDa predicted using DNAStar. In order to confirm the result and assure the correctness of ORF prediction, further western blot analysis was performed with RGV-infected cells, 50L-pcDNA3.1 transfected cells, purified RGV particles and control cells. As shown in Fig. 3, positive signals could be detected in RGV-infected cells, 50L-pcDNA3.1 transfected cells and purified RGV particles (Lane 3–5 respectively), and the positive bands were about 85 kDa, while no signals were detected in mock-infected cells and pcDNA3.1 transfected cells (Lane 1 and 2 respectively). The result confirmed that the MW of RGV 50L was about 85 kDa and the predicted ORF was correct.

**Figure 6:**
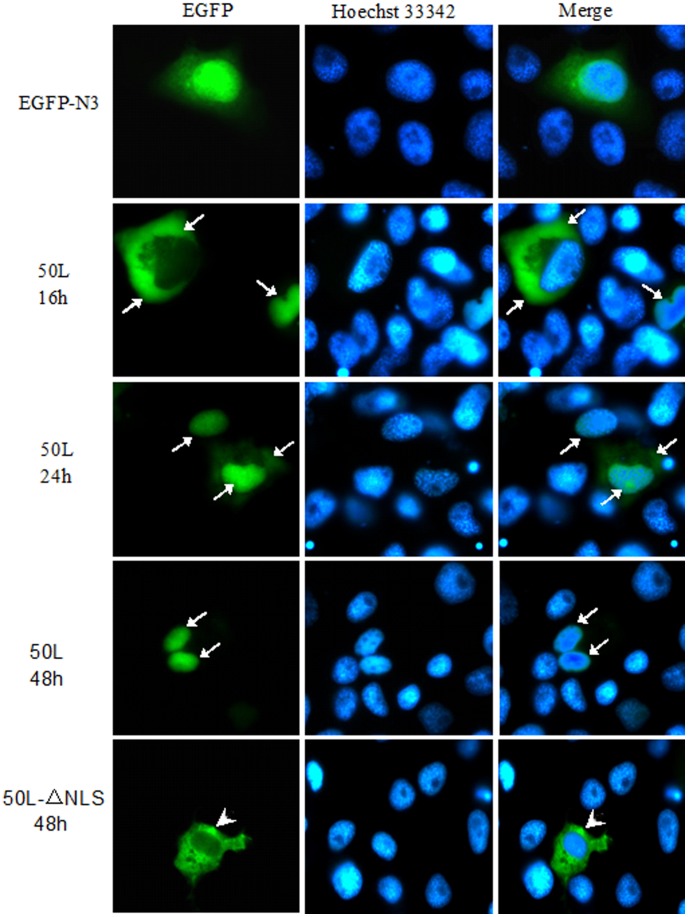
Subcellular localization of 50L detected by 50L-EGFP fusion protein. First EPC cells were transfected with plasmid pEGFP-50L or pEGFP-N3 and the fusion proteins were detected at 16 h and 24 h. Then the cells were transfected with plasmid pEGFP-50L or pEGFP-50L-ΔNLS and the fusion proteins were detected at 48 h. Green fluorescence showed the localization of the fusion protein (EGFP), the cell nuclei were shown by Hoechst 33342 (Hoechst 33342), and the merged photos were also listed (Merged). 50L-EGFP fusion protein was indicated by long arrows and 50L-ΔNLS-EGFP protein was indicated by short arrows. Magnification ×100 (oil-immersion objective).

### Identification of RGV 50L as an Immediate-early Gene

To verify the transcriptional pattern of RGV, drug inhibition assay was carried out using Cycloheximide (CHX) and Cytosine β-D-arabinofuranoside (Ara C). The samples were detected by RT-PCR and western blot analysis, and the 50L, dUTPase and MCP were confirmed to be IE, E and L transcripts gene, respectively. As shown in Fig. 4A, the 50L transcript could be detected in the RGV-infected samples and the samples treated with 50 µg ml^−1^ of CHX and infected with RGV for 6 h, and that treated with 100 µg ml^−1^ of Ara C and infected with RGV for 48 h, but not in samples only treated drugs above. Proteins extracted from the corresponding samples were detected by western blot analysis. The result was shown in Fig. 4B, which was consistent with RT-PCR analysis. The data demonstrated that RGV 50L is an IE gene during the *in vitro* infection.

**Figure 7:**
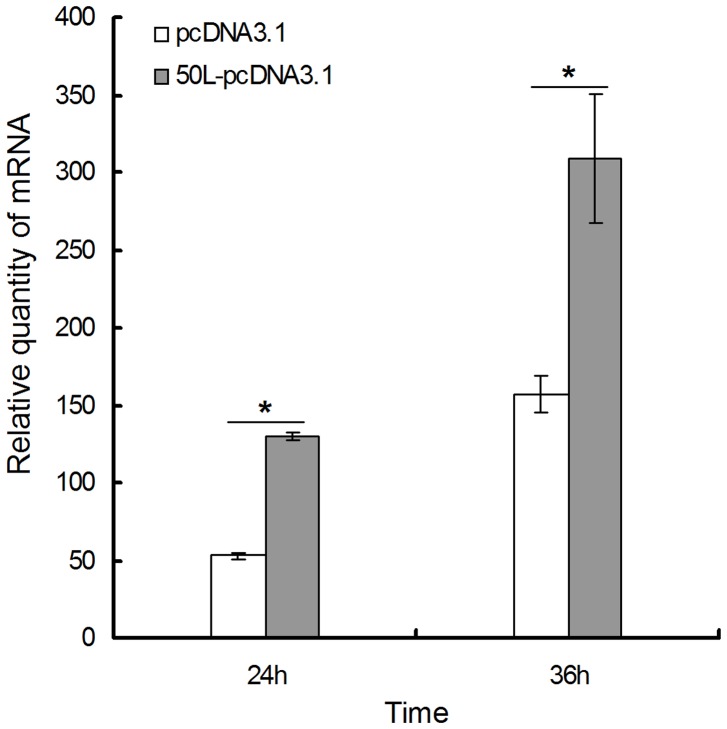
Effect of 50L over-expression on mRNA level of RGV *53R*. EPC cells were transfected with pcDNA3.1 or pcDNA3.1-50L. Then, after 24 h, the transfectants were mock-infected or infected by 1 M.O.I. of RGV respectively. Total RNAs were extracted at 24 and 36 h p.i. mRNA level of RGV *53R* gene was detected by qRT-PCR. Relative quantities for each sample were expressed as N-fold changes in target gene expression relative to the same gene target in the calibrator sample, and normalized to the β-actin gene. The values represent averages of three independent experiments, with the range indicated (±SD). The significant differences between control and treatments groups are determined by T-TEST. *p<0.001.

### Intracellular Localization of RGV 50L

Immuno-fluorescence assay was performed to reveal the intracellular localizations of 50L distribution. ΔTK-RGV, which could emit green fluorescence, was used to confirm the infection of RGV. As shown in Fig. 5, 50L appeared early and persisted in the infected cells, and its localization changes of 50L followed two routes, one route was that weak red signals could be detected initially in the cytoplasm at 6 h post infection (p.i.), later appeared in both the cytoplasm and nucleolus at 8 h p.i, then mainly in the nucleolus at 10 h p.i. and the phenomenon was similar at 12 h p.i., subsequently, the RGV-infected cells were observed to be in clusters and strong signals could be detected in the cytoplasm, nucleus and viral matrix at 16 h p.i., at last, the signals aggregated mainly in the viral matrix; the other was that 50L co-localized with viral matrix (arrows): at first the viral matrix was very tiny, and the red fluorescent signal of 50L was a tiny spot, then viral matrices became bigger and bigger, and the red spotted signals of 50L also increased, at last the viral matrix became a large one near the nucleus and completely co-localized with 50L.

**Figure 8:**
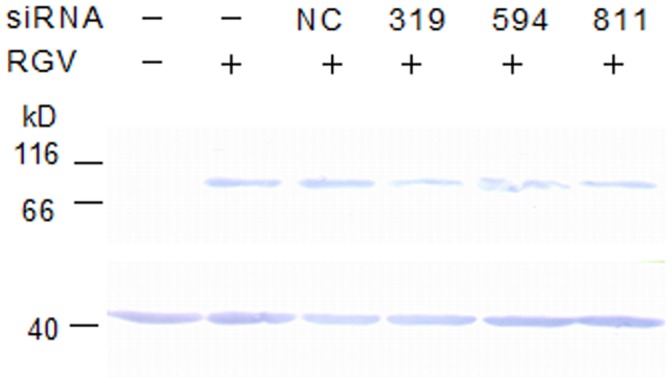
RGV 50L silencing assay by siRNA. EPC cells, cultured in 24-well plates at about 8.0×10^5^ cells/ml, were transfected with siRNAs targeted to *50L* (siRNA-319, 594 and 811) and a negative control (siRNA-NC) at a final concentration of 150 nM, respectively. Then, 5 h later, the transfected and un-transfected cells were incubated with approximately 1 MOI RGV for 1 h and harvested at 24 h p.i. The silence effect of siRNAs on the expression of 50L was detected by western blot analysis. β-actin gene was used as an internal control.

Dynamic changes of 50L-EGFP fusion protein in pEGFP-50L-transfected cell were detected. Strong green fluorescent signals (long arrows) first appeared mainly in the cytoplasm and little in the nucleus at 16 h after transfection, then less in the cytoplasm and more in the nucleus at 24 h, and only in the nucleus at 48 h (Fig. 6). Furthermore, in the site-directed mutagenesis assay, normal *50L* and NLS mutant *50L* were used for transfection, and green fluorescence was detected at 48 h after transfection. The results showed that green fluorescent signals only appeared in the nucleus in the pEGFP-50L transfected cells, however, positive signals (short arrows) only appeared in the cytoplasm of the pEGFP-50L-ΔNLS transfected cells at 48 h (Fig. 6), which suggested that the NLS motif of RGV 50L plays an important role in its localization in the nucleus of cells.

**Table 2:** Summary of the putative post translational modifications in 50L protein.

Motif	Site	Pattern	Randomized probability
cAMP- and cGMP- dependent protein kinasephosphorylation site	339 to 342 KRRT	[RK](2)-x-[ST]	1.572e−03
Protein kinase C phosphorylation site	70 to 72 SKK	[ST]-x-[RK]	1.423e−02
	180 to182 TAK		
	376 to378 TLK		
	422 to424 TKR.		
Casein kinase II phosphorylation site	35 to 38 TFSE	[ST]-x(2)-[DE]	1.482e−02
	78 to 81 SYAD		
	100 to 103 SEPE		
	189 to 192 TKTE		
	191 to 194 TESE		
	227 to 230 SDSE		
	229 to 232 SENE		
	289 to 292 TADD		
	293 to 296 SSDD		
	325 to 328 SDSE		
	327 to 330 SEAE		
	349 to 352 SSDD		
	350 to 353 SDDE		
	429 to 432 SKVD		
	492 to 495 SWTE		
N-myristoylation site	23 to 28 GLGHTM	G-{EDRKHPFYW}-x(2)-[STAGCN]-{P}	1.397e−02
	246 to 251 GVRKTM		
	379 to 384 GMCKTR		
	390 to 395 GNKAAL		
	488 to 493 GIRYSW		

### Effects of 50L on mRNA Levels of RGV *53R*

RGV 53R is an important structural protein of RGV. The effect of RGV 50L on the transcriptional level of the gene was analyzed by qRT-PCR, which detected the relative mRNA level of *53R* in the 50L-pcDNA3.1/pcDNA3.1 transfected cells after infected by RGV. Compared with the controls (pcDNA3.1 transfected cells), mRNA level of *53R* was higher at 24 and 36 h p.i. (Fig. 7). The result implied that 50L protein may effect the transcription of RGV*53R*.

**Table 3:** Primers used for plasmid construction, RT-PCR and quantity real time PCR.

Primer name	Sequence (5′-3′) (enzyme cleavage site was underlined)
50L-pro-F	ATT*GGATCC*ATGCAAGTCTACTCTCC (*BamHI*)
50L-pro-R	*AAGCTT* GTAACACAGATAATCTTCAG (*HindIII*)
50L-GFP-F	ATT*GCTAGC*ATGCAAGTCTACTCTCC (*NheI*)
50L-GFP-R	CAA*GGATCC*CTCACACAGATAATCTTC (*BamHI*)
50L-3.1-F	ATT*GCTAGC*ATGGAAGTCTACTCTCC (*NheI*)
50L-3.1-R	CAAA*GGATCC*TAACACAGATAATCTTC (*BamHI*)
50L-ΔNSL-F	GCCTGTAGAGCAGCCTACAGCCGTTAGAAAGTCTAGAGCAAA
50L-ΔNSL-R	TTTGCTCTAGACTTTCTAACGGCTGTAGGCTGCTCTACAGGC
50L-RT-F	GGCAGAGAGCACATGCTGATGG
50L-RT-R	GTCCAGCTGTACCTGATGCCCATG
DUT-RT-F	TGGTCCCCTCCTTTGGCAG
DUT-RT-R	ACCCCTGTCGGTAGAGTCCA
MCP-RT-F	GACTTGGCCACTTATGAC
MCP-RT-R	GTCTCTGGAGAAGAAGAA
50L-qRT-F	AAAAGCTGGACGAGGCTACA
50L-qRT-R	AGCTATGCCGTCTGCCTCTA
53R-qRT-F	CCAAGGTCACCATGACACAG
53R-qRT-R	CCAGAACGATGATGACGATG
β-actin-F	CACTGTGCCCATCTACGAG
β-actin-R	CCATCTCCTGCTCGAAGTC

Note: Restriction sites are italicized.

### Effect of siRNAs on RGV *50L* Silencing

To knockdown RGV *50L*, three chemically synthesized siRNAs targeted to *50L* and a negative control siRNA were used to reduce *50L* gene expression. As shown in Fig. 8, the band of 50L in the siRNA-319 transfected sample was the weakest at 24 h p.i., which revealed that siRNA-319 suppressed the expression of RGV 50L most effectively among the four siRNAs. So siRNA-319 was selected for viral titer assay (expressed as TCID_50_/ml). Viral titers of siRNA-NC and un-transfected samples did not show significant difference with that of siRNA-319 transfected samples, and cytopathic effects were similar among these samples (data not shown).

**Table 4:** siRNA sequences (sense strand) used in this study.

siRNA name	Target sequence	Position in gene sequence
si50L594	GGCUUGACCUCGUUGUAAUTT	594–614
si50L811	CAGAGUCGCUUAUAACAAATT	811–831
si50L319	CGGCCUUGUUUCCAGAUAUTT	319–339
siNC	UUCUCCGAACGUGUCACGUTT	

## Discussion

Although homologues of RGV 50L could be found in many iridoviruses belonging to the genera *Ranavirus* and *Lymphocystivirus*, those from lymphocystiviruses showed low identities with 50L, and the predicted molecular masses and identity percentages compared with 50L of those from other ranaviruses made a great difference, which may be related to different adaptabilities of different viruses.

The MW of 50L detected by western blot assay in RGV infected cells was 85 kDa, which was much larger than the predicted 55 kDa. Further western blot analysis of RGV-infected cells, pcDNA3.1–50L transfected cells and purified RGV particles showed that the positive bands were about 85 kDa and identical in the three samples (which was consisted with our previous report that RGV indeed contains a 85 kDa structural protein [2]). The data confirmed that the protein encoded by 50L gene was actually larger than the predicted data, and also demonstrated that the ORF prediction of 50L was correct. The difference in the actual and predicted MW suggested that RGV 50L may be subjected to eukaryotic post translational modifications, which are indispensable for functions of some proteins [27], [28]. This is in line with the characteristic of 50L sequence. PredictProtein analysis of 50L sequence showed that it contained many putative phosphorylation sites besides five N-myristoylation sites, such as one cAMP- and cGMP-dependent protein kinase phosphorylation site, four protein kinase C phosphorylation sites and fourteen casein kinase II phosphorylation sites (Table 2). Similar phenomena have been observed in Singapore grouper iridovirus (SGIV) ICP18 and ICP46 [29], [30].

The iridovirus genes are expressed in three temporal kinetic classes: immediate-early (IE), early (E) or delayed-early (DE) and late (L) during the viral infection, which can be defined by *de novo* viral protein synthesis and DNA replication inhibitors [31], [32]. Drug inhibition assay showed that 50L could not be inhibited by CHX or Ara C, suggesting that it was an IE gene, and the transcriptional pattern of which was identical to that of 3β-HSD an IE gene identified previously [6]. Many researches on large DNA viruses infecting mammals have been reported [33]–[35], but studies on characteristics of iridovirus IE genes are rare.

Intracellular localizations of 50L during RGV-infection detected by immuno-fluorescent assay revealed that location changes of 50L followed two patterns. One pattern was that 50L exhibited a cytoplasm-nucleus-vitromatrix distribution pattern, and the other was that 50L co-localized with viral matrix, which has not been reported in iridoviruses to date. Proteins are synthesized in the cytoplasm, so it was not surprising that 50L presented in the cytoplasm. However, it is believed that only ions and small molecules (relative molecular mass less than 40–60 kDa) are freely permeable to the nuclear pore complex (NPC), and macromolecules were imported by energy-dependent mechanisms [36], [37]. As stated in the above, the MW of 50L is about 85 kDa, which is too large to be translocated trough the NPC. 50L-EGFP fusion protein could also translocate from the cytoplasm to the nucleus in pEGFP-50L transfected cells, but how did it enter into the nucleus? A lysine-rich NLS was predicted at the N-terminus of the RGV 50L, and the NLS deleted mutation experiment showed that the normal 50L containing an NLS motif could be imported into the nucleus successfully, while the mutant 50L without an NLS could not be imported into the nucleus and was diffuse in the cytoplasm. The results revealed that nucleus translocation of 50L was NLS-dependent, as NLS could import macromolecular cargoes into the nucleus by binding to nuclear transport proteins through the nuclear pore [38]. The exportation of 50L from the nucleus to the cytoplasm may be related to the putative leucine-rich nuclear-exported signal (NES) motif formed by residuals 384–394, which could export macromolecules from the nucleus to the cytoplasm [39], [40]. But how the exportation actually took place needs further investigation.

Viral matrix, the place for virus assembly, contains viral DNA, large quantities of virus structural proteins and other components [14], [41]. In this study, a part of 50L was detected to accompany the viral matrix: the signals of 50L were very weak at first, then it gradually increased with the enlargement of viral matrices. This phenomenon was consisted with previous electron microscopy studies of NCLDVs-infected cells, which showed that small low density viral matrix formed in the cytoplasm as early as 3 h p.i., the size of which increased with time and with the production of progeny virions [5], [42]. As shown in Table 2, 50L was predicted to contain five putative M-G-X-X-X-(S/T/A) N-myristoylation sites, which were shown to be required for the assembly of many viruses [43], [44]. Furthermore, 50L, as a virus structural protein, appeared early and persisted in the cells till the late stage of infection, so it is no doubt that 50L plays an important role in RGV assembly and life circle.

Effects of RGV 50L on mRNA levels of RGV *53R* detected by qRT-PCR showed that 50L could affect the transcriptional level of the important structural protein encoding gene. Second structure of 50L was predicted to contain a glutamine and glutamic acid-rich tri-repeated domain in the N-terminus and a SAP domain in the C-terminus which was proved to be a new type of eukaryotic DNA binding domain and associated in gene transcription [45], [46]. Whether the effect of 50L on the gene is related to these structures and whether 50L could effect transcriptions of other genes need further studies.

Furthermore, expression of RGV 50L could be reduced by siRNA-319. However, the virus yields of offspring did not show significant difference among the siRNA-319, siRNA-NC and un-transfected samples. This result may implicate that RGV 50L is not a gene associated with virus replication directly *in* *vitro*. Similar phenomenon was also observed in another ranavirus IE gene FV3 ICP18 knocked down using antisense morpholino oligonucleotides (asMOs) [47]. Our findings suggested that 50L is directly related to virus assembly and implied that RGV 50L may contribute indirectly to ranavirus replication by affecting the expression of other structural proteins. Additionally, it is also possible that as the expression of RGV 50L was not inhibited completely by siRNA, a small quantity of RGV 50L may be enough for RGV replication. However, how RGV 50L exactly works still needs further studies.

In conclusion, we have cloned and characterized RGV *50L* gene as an IE gene of RGV, and revealed that 50L appeared early and persisted in RGV-infected cells following two distribution patterns, one pattern was that 50L exhibited a cytoplasm-nucleus-viromatrix distribution pattern, and the other was that 50L co-localized with viral matrix. This phenomenon was the first report in iridoviruses. The data reveals that RGV *50L* is a novel IE gene encoding a virus structural protein associated with virus assembly.

## Materials and Methods

### Virus and Cells

RGV was used in this study. *Epithelioma papulosum cyprinid* (EPC) cells grown in TC 199 medium supplemented with 10% fetal bovine serum (FBS) at 25°C were used for virus propagation. Cell culture, virus propagation and DNA purification were performed as we described previously [2], [48].

### Gene Cloning, Protein Sequence Analysis and Plasmids Construction

The full length of RGV 50L was amplified from genomic DNA with specific primers containing restriction enzyme cleavage sites, respectively (Table 3). PCR was carried out under the following conditions: 4 min at 94°C and then 30 s at 94°C, 30 s at 56°C, 1.5 min at 72°C for 32 cycles, followed by 72°C for 10 min. The amplified fragments were cloned into prokaryotic vector pET32a (+), eukaryotic vector pEGFP-N3 and pcDNA3.1 (+) by corresponding restriction enzymes respectively. These different constructs were named pET32a-50L, pEGFP-50L and pcDNA3.1-50L, respectively. All the constructs were confirmed by restriction enzyme digestion and DNA sequencing.

The sequence data were compiled and analyzed using DNASTAR software. The non-redundant protein sequence database of the National Center for Biotechnology Information (National Institutes of Health, MD, USA) was searched using BLASTP. Multiple sequence alignments were conducted using CLUSTAL_X v1.83 and edited using GeneDoc. Further patterns/signatures and structure analysis of 50L amino acid sequence were carried out by Network Protein Sequence Analysis server (NPS@ server) [49], and NLS was predicted using PredictProtein server (https://www.Predict protein.org) [50]. NES prediction of RGV 50L was performed using the CBS online service NetNES 1.1 (http://www.cbs.dtu.dk/services/NetNES) [51].

NLS coding sequence was removed by site-directed mutagenesis using an overlap extension-PCR method in a two-step PCR procedure [52]. Briefly, in the first step, two simultaneous PCR reactions were performed. One reaction was performed with primers 50L-EGFP-F and 50L-ΔNLS-R to amplify the N-terminal of the 50L, the other reaction was performed with primers 50L-ΔNLS-F and 50L-EGFP-R to amplify the C-terminal. To obtain the full-length mutated fragment without the NLS, equal amounts of the two products from the first step were mixed and used as templates for the second PCR reaction, with primers 50L-EGFP-F and 50L-EGFP-R. Finally, the full length mutated fragments were ligated into pEGFP-N3 vector, and the construct was named EGFP-50L-ΔNLS.

### Prokaryotic Expression, Protein Purification and Antibody Preparation

50L-His fusion protein expression, purification and antibody preparation were performed as we previously described [24]. Briefly, 50L-pro plasmid was induced with 1 mM IPTG at 37°C to express the recombinant protein after transformed into *Escherichia coli* BL21 (DE3). The recombinant protein was purified according to the protocols of the HisBind Purification Kit (Novagen). To obtain antibody of RGV 50L, the purified fusion protein (about 400 µg) was mixed with equal volume of Freund’s adjuvant (Sigma) to immunize mice once every 7 days, and the antiserum was collected after the fourth immunization.

This experiment was carried out in strict accordance with the recommendations in the Regulations for the Administration of Affairs Concerning Experimental Animals of China. The protocol was approved by the Wuhan University Center for Animal Experiment (Approval ID: SCXK 2008-0004). All surgery was performed under sodium pentobarbital anesthesia, and all efforts were made to minimize suffering.

### Real-time Quantitative PCR and Western Blot Analysis of 50L Temporal Expression

Total RNAs and protein were prepared from cells infected by RGV at an M.O.I. of 1 at various time (0, 4, 8, 12, 16, 24, 36 and 48 h) post-infection (p.i.) or mock infected, and subjected to real-time quantitative PCR and western blot analysis, respectively. The synthesis of cDNA was carried out as described previously [11]. Real-time quantitative PCR was performed with Fast SYBR® Green Master Mix using the StepOne™ Real-Time PCR System (Applied Biosystems Ins., USA). Each reaction consisted of 1 µl of product from the diluted RT reaction, 10 µl 2×Fast SYBR® Green Master Mix, 250 nM of sense and antisense primer and sterile water. The mixture was incubated in a 48-well plate at 95°C for 20 sec, followed by 40 cycles of 95°C for 3 sec and 60°C for 30 sec. The melting curve analysis of PCR products from 60°C to 95°C were performed after PCR. Primers were named 50L-qRT-F/50L-qRT-R, 53R-qRT-F/53R-qRT-R and MCP-qRT-F/MCP- qRT-R, respectively (Table 1). For relative quantification of each sample, the relative standard curve quantification method was employed, and all experimental data were normalized to the β-actin gene. The data were expressed as means±SD from three independent experiments.

Western blot analysis was carried out as described previously [53]. Briefly, protein samples prepared above were electrophoresed in 12% SDS-PAGE and transferred to a PVDF membrane (Millipore). The membrane was blocked with 5% skim milk in TBS (0.02 M Tris–HCl pH 7.4; 154 mM NaCl) for 1 h at room temperature. Then, the membrane was e incubated successively with 1∶1000 diluted RGV 50L mouse anti-serum for 2 h, and 1∶1000 diluted alkaline phosphatase-conjugated goat anti-mouse IgG (H+L) antibody (Vector Laboratories) for 1 h. Finally, substrates NBT and BCIP (Sigma, USA) were used for color reaction. Internal control was carried out simultaneously by detecting β-actin protein.

### Molecular Weight Identification of 50L

Western blot assay was applied to identify the molecular weight of 50L in eukaryotic cells. Plasmid pcDNA3.1-50L/pcDNA3.1 was transfected into EPC cells by Lipofectamine® 2000 Reagent (Invitrogen) following the instructions, and the samples were subjected to western blot analysis after incubated for 12 h. Mock- and RGV-infected cells at 12 h p.i. and purified RGV particles were analyzed together. Procedures for western blot were carried out as described above.

### Drug Inhibition of *de novo* Protein Synthesis and Viral DNA Replication

Cycloheximide (CHX), as *de novo* protein synthesis inhibitor, and Cytosine β-D- arabinofuranoside (Ara C), as viral DNA replication inhibitor, were selected to classify the transcriptional model of RGV 50L. The experiment and RT-PCR analysis were carried out as described previously [8]. Specific primers were used to detect RGV 50L transcripts (50L-RT-F/50L-RT-R in Table 1). As control, two pairs of primers were used to detect the transcripts of the known early (E) transcription gene, dUTPase, and late (L) transcription gene, major capsid protein (MCP), respectively (primers DUT-RT-F/DUT-RT-R and MCP-RT-F/MCP-RT-R in Table 1) [8], [19]. β-actin was also performed as internal control. Protein from each sample was extracted and western blot analysis was carried out as described above.

### Subcellular Localization

Subcellular localization of RGV 50L was performed by 50L-EGFP fusion protein expression and immunoﬂuorescence. For EGFP fusion protein expression, EPC cells were cultured on coverslips in 6-well plates and transfected with plasmid pEGFP-50L, and plasmid pEGFP-N3 was used as control. After 16 h and 24 h incubation, the cells were fixed and stained with Hoechst 33342 in PBS for 5 min at room temperature.

To examine the effect of the NLS motif on RGV 50L translocation, the recombinant plasmids pEGFP-50L and pEGFP-50L-ΔNLS were used to track the movement of normal or mutant RGV 50L-EGFP fusion protein. The cells were fixed and stained as describe above at 48 h after transfection.

In order to observe the intracellular localization of 50L during RGV-infection, immuno- ﬂuorescence microscopy was carried out as previously described [54]. EPC cells, grown on coverslips in 6-well plates, were either mock or infected with approximately 1 MOI RGV and fixed at 6 h, 8 h, 10 h, 12 h, 16 h and 24 h. After blocked in 10% bovine serum albumin at room temperature for 1 h, the cells were then successively incubated with mice anti-RGV-50L serum in 1% normal bovine serum and Rhodamine Red-X Goat Anti-Mouse IgG (Pierce Biotechnology, Rockford). Nuclei were counterstained with Hoechst-33342. All samples were examined under a Leica DM IRB ﬂuorescence microscope.

### Effects of 50L Over-expression on Transcriptional Level of RGV *53R*

About 8.0×10^5^ EPC cells were seeded into 24-well plates and transfected with empty vector pcDNA3.1 or pcDNA3.1-50L using the method mentioned above after 16 h. The pcDNA3.1 and pcDNA3.1-50L-transfected EPC cells were termed as pcDNA3.1/EPC and 50L-pcDNA3.1/EPC, respectively. Following transfection for 24 h, the transfectants were mock-infected or infected by RGV at an M.O.I. of 1. Total RNAs were extracted at 24 and 36 h p.i., and mock infected cells were used as control. Subsequent real-time quantitative PCR for RGV *53R* was done as above using primers 53R-qRT-F and 53R-qRT-R (Table 3) and relative quantities for each sample were expressed as N-fold changes in target gene expression relative to the same gene target in the calibrator sample, both normalized to the β-actin gene. The significant differences between control and treatments groups are determined by T-TEST.

### Knockdown of *50L* Expression by RNAi

Three duplex siRNAs targeted to *50L* and a negative control (siNC) (Table 4, GenePharma, Shanghai, China) were chemically synthesized for knockdown of RGV *50L*, and the experiment was carried out as described previously with some modifications [10], [23]. Briefly, EPC cells were cultured in 24-well plates at a density of about 8.0×10^5^ cells/ml. The siRNAs were transfected at a final concentration of 150 nM, respectively. Then, the un-transfected and transfected cells were infected with approximately 1 MOI RGV after 5 h and incubated 1 h at 25°C, and the samples were mixed gently every 15 min and harvested at 24 h p.i. The silence effect of siRNAs was detected by western blot analysis, and un-treated cells were used as negative control. Subsequently, EPC cells were un-transfected or transfected with the siRNA with silence effect or siRNA-NC. RGV infection was carried out as described above, and every sample was triplicates. Finally, all samples were serially diluted 10-fold after three cycles of freeze-thaw, and 100 µL of each dilution was added to four repetitive wells of confluent EPC monolayers grown on 96-well plates to perform the TCID_50_ assay.
